# Sirt6 Mono‐ADP‐Ribosylates YY1 to Promote Dystrophin Expression for Neuromuscular Transmission

**DOI:** 10.1002/advs.202406390

**Published:** 2024-10-10

**Authors:** Wei Zhang, Lei Bai, Wentao Xu, Jun Liu, Yi Chen, Weiqiang Lin, Huasong Lu, Binwei Wang, Benyan Luo, Guoping Peng, Kejing Zhang, Chengyong Shen

**Affiliations:** ^1^ Department of Neurobiology of First Affiliated Hospital Zhejiang Key Laboratory of Frontier Medical Research on Cancer Metabolism Institute of Translational Medicine School of Medicine Zhejiang University Hangzhou China; ^2^ Department of Pharmacology Nanjing University of Chinese Medicine Nanjing China; ^3^ Department of Nephrology Center for Regeneration and Aging Medicine The Fourth Affiliated Hospital of School of Medicine and International School of Medicine International Institutes of Medicine Zhejiang University Yiwu China; ^4^ Life Sciences Institute Zhejiang University Hangzhou China; ^5^ Department of Neurobiology First Affiliated Hospital School of Medicine Zhejiang University Hangzhou China; ^6^ Zhejiang Provincial Key Laboratory of Pancreatic Disease MOE Joint International Research Laboratory of Pancreatic Diseases First Affiliated Hospital Hangzhou 310006 China; ^7^ MOE Frontier Science Center for Brain Research and Brain‐Machine Integration Zhejiang University Nanhu Brain‐Computer Interface Institute Hangzhou China

**Keywords:** Dystrophin, NMJ, Sirt6, YY1

## Abstract

The degeneration of the neuromuscular junction (NMJ) and the decline in motor function are common features of aging, but the underlying mechanisms have remained largely unclear. This study reveals that Sirt6 is reduced in aged mouse muscles. Ablation of Sirt6 in skeletal muscle causes a reduction of Dystrophin levels, resulting in premature NMJ degeneration, compromised neuromuscular transmission, and a deterioration in motor performance. Mechanistic studies show that Sirt6 negatively regulates the stability of the Dystrophin repressor YY1 (Yin Yang 1). Specifically, Sirt6 mono‐ADP‐ribosylates YY1, causing its disassociation from the Dystrophin promoter and allowing YY1 to bind to the SMURF2 E3 ligase, leading to its degradation. Importantly, supplementation with nicotinamide mononucleotide (NMN) enhances the mono‐ADP‐ribosylation of YY1 and effectively delays NMJ degeneration and the decline in motor function in elderly mice. These findings provide valuable insights into the intricate mechanisms underlying NMJ degeneration during aging. Targeting Sirt6 could be a potential therapeutic approach to mitigate the detrimental effects on NMJ degeneration and improve motor function in the elderly population.

## 1 Introduction

Aging, an inevitable process for all adults, often results in diminished motor functions, such as unstable gait, weakness, and even the inability to carry out daily activities. Skeletal muscle contraction and movement are controlled by motor neurons via the neuromuscular junctions (NMJs), which are the synaptic connections between motor neurons and muscle fibers. Agrin‐Lrp4‐MuSK‐Dok7‐Rapsyn‐AChR signaling is critical for AChR clustering and NMJ formation.^[^ ^1^ ^]^ As aging progresses, NMJs undergo degeneration in muscles, leading to decreased efficiency in neuromuscular synaptic transmission and consequently, weakened motor functions.^[^ ^1^ ^,^ ^2^ ^]^ The mechanisms underlying NMJ degeneration in the elderly remain largely unknown.

The Dystrophin‐Glycoprotein complex (DGC) plays a pivotal role in maintaining the structure and function of NMJs.^[^ ^1^ ^,^ ^3^ ^]^ Interestingly, the presence of DGC components, including Dystrophin, in the NMJ region is higher than in other regions along the muscle fiber.^[^ ^3^ ^]^ NMJs can form in mice without Dystrophin and its homolog Utrophin, but they fail to maintain their normal structure.^[^ ^4^ ^]^ The postsynaptic assembly of AChR clusters undergoes fragmentation and degeneration, leading to muscle weakness. Furthermore, mutations in Dystrophin cause Duchenne muscular dystrophy (DMD). DMD patients exhibit muscle weakness and NMJ fragmentation and degeneration.^[^ ^5^ ^,^ ^6^ ^]^ So far, the role and mechanisms of Dystrophin in NMJ degeneration in aged muscles are unclear.

The Sirtuin family, closely associated with aging, comprises seven members (Sirtuin 1–7) in humans and mice. Among these, Sirt6, primarily functioning in the cell nucleus, has been found to have a strong correlation with aging.^[^ ^7^ ^,^ ^8^ ^]^ Sirt6 exhibits nicotinamide adenine dinucleotide (NAD^+^)‐dependent deacetylation, defatty‐acylation, and mono‐ADP‐ribosylation activities.^[^ ^8^ ^‐^ ^10^ ^]^ NAD^+^ in cells could be converted from its precursor, nicotinamide mononucleotide (NMN).^[^ ^11^ ^]^ Animals deficient in Sirt6 display premature aging phenotypes, such as a small body size, thin subcutaneous fat layer, muscle atrophy, and weakness, with significantly shortened lifespan.^[^ ^12^ ^,^ ^13^ ^]^ In contrast, transgenic mice with overexpression of Sirt6 show extended lifespan.^[^ ^14^ ^,^ ^15^ ^]^ Notably, Sirt6 expression and activity in humans and mice have been reported to decline with age.^[^ ^8^ ^,^ ^11^ ^,^ ^16^ ^‐^ ^18^ ^]^ Sirt6 was reported to play multiple roles in muscles^[^ ^19^ ^‐^ ^22^ ^]^; however, the link between the decline of Sirt6 and age‐related NMJ degeneration in muscles remains unexplored. It is also unclear whether enhancing Sirt6 activity can inhibit NMJ degeneration and improve motor functions.

In this study, we have unveiled that the absence of Sirt6 in mouse skeletal muscles leads to a reduction of Dystrophin levels, resulting in premature NMJ degeneration, compromised neuromuscular transmission, and a deterioration in motor performance. Mechanistic studies found that Sirt6 mono‐ADP‐ribosylates YY1, which prompts its disassociation from the Dystrophin promoter and subsequent binding to the SMURF2 E3 ligase, leading to its degradation. Importantly, the supplementation of NMN enhances the mono‐ADP‐ribosylation of YY1 and effectively delays NMJ degeneration and the decline in motor function in elderly mice. Our findings provide valuable insights underlying NMJ degeneration during aging, and evaluate a potentially therapeutic approach to improve motor function in the elderly population.

## 2 Methods

### 2.1 Animals

Sirt6^flox/flox^ mice (No.017334) and HSA‐Cre transgenic mice (No.006149) were obtained from Jackson Laboratories, USA. Sirt6^flox/flox^ mice were bred with HSA‐Cre mice to generate skeletal muscle‐specific Sirt6 knockout mice (HSA‐Sirt6 cKO). For behavioral analysis, only male mice were used. In other experiments, either male or female pair of littermates (control and HSA‐Sirt6 cKO mice) were used. All mice were of the C57BL/6J background, and were maintained under standard conditions and a 12‐hour light and 12‐hour dark cycle with free access to food and water. Animal experimental procedures were approved by the Institutional Animal Care and Use Committee of Zhejiang University (20 211 046).

### 2.2 Plasmids

The Sirt6‐HA and SMURF2‐Myc cDNAs were obtained from Miaoling Bio and YY1‐Flag from Transheep Bio. YY1 cDNA was cloned into the pCMV vector using the Bgl II and Mlu I restriction sites. The YY1‐E216A/E218A/K409A mutant and Sirt6 mutants (Sirt6‐S56Y, Sirt6‐G60A, Sirt6‐R65A, and Sirt6‐H133Y) were generated by site‐directed mutagenesis. The single guide RNA targeting Sirt6 (sgSirt6, AATGTGGCAGTCCTCCAGCG) and YY1 (sgYY1, CGACCCGGGGAATAAGAAGT) were inserted into the pLentiCRISPR v2 vector (#52 961, Addgene).

### 2.3 Cell Culture and Treatment

Mouse C2C12 myoblast cells were cultured in DMEM supplemented with 20% FBS and 100 units/ml penicillin and streptomycin. To induce myoblast differentiation into myotubes, cells were cultured in DMEM containing 2% horse serum as previously described.^[^ ^23^ ^]^ The following compounds were used for treatment: MG132 (10 µM, 2 hours), chloroquine (CQ, 10 µM, 2 hours), cycloheximide (CHX, 10 µM, for the indicated hours), the Sirt6 inhibitor OSS‐128167 (Sirt6i, 100 µM, overnight), the Sirt6 agonist MDL‐800 (Sirt6a, 10 µM, overnight), or NAD^+^ (100 µM, overnight). These chemicals were obtained from MCE or Sigma.

Lentiviruses were produced by co‐transfecting HEK293T cells with the pLentiCRISPR v2, pMD2.G (#12 259, Addgene), and psPAX2 (#12 260, Addgene) plasmids. To generate Sirt6 knockout (KO) cells, C2C12 cells were infected with lentiviruses expressing sgSirt6 for 48 hours before selection with 2 µg/ml puromycin. The efficiency of Sirt6 knockout was analyzed by Westernblot. To generate Sirt6 and YY1 double KO cells, sgYY1 were cloned into a modified pLentiCRISPR v2 vector containing GFP. Sirt6 KO cells were infected with lentiviruses expressing sgYY1, followed by FACS using a flow cytometer (BD FACSAria III).

### 2.4 Isolation of Synaptic Region (SR)

Mice were sacrificed, and diaphragm muscles were promptly isolated as previously described.^[^ ^24^ ^]^ The muscles were then stained with Rhodamine‐conjugated α‐Bungarotoxin (R‐BTX; 1:5000; Life Sciences) for 5 minutes in cold PBS. Using a scalpel, two flanks of NMJ region were carefully separated under a stereo fluorescence microscope (SMZ18, Nikon). The BTX‐positive region, which represents the synaptic region (SR), was distinguished from the BTX‐negative region (the non‐synaptic region, NSR). The samples were stored in liquid nitrogen for subsequent immunoblot analysis.

### 2.5 Immunoprecipitation and Immunoblot

Immunoprecipitation (IP) and immunoblot was performed as previously described.^[^ ^25^ ^,^ ^26^ ^]^ For IP analysis, cells were lysed in a lysis buffer containing 50 mM Tris‐HCl (pH 7.6), 150 mM NaCl, 1% NP40, 2.5 mM EDTA, 1 mM PMSF, and protease inhibitor cocktails (HY‐K0010, MCE). The lysates were then centrifuged at 14 000 g for 15 minutes. The supernatants were collected and incubated overnight with the indicated antibodies and protein A/G agarose beads (sc2003, Santa Cruz) or anti‐Flag beads (P2282, Beyotime). The beads were washed five times with IP lysis buffer and incubated with SDS loading buffer at 95 °C for 5 min and then subjected to immunoblot.

For immunoblotting, the following primary antibodies were used: anti‐Sirt6 (1:1000, 12486S, Cell Signaling Technology), anti‐Neurofilament (1:1000, 2837, Cell Signaling Technology), anti‐Dystrophin (1:100, ab1527, Abcam), anti‐YY1 (1:100, 22156‐1‐AP, Proteintech; 1:500, 66281‐1‐Ig, Proteintech); anti‐SMURF2 (1:1000, A10592, Abclonal), anti‐Ubiquitin (1:1000, sc‐8017, Santa Cruz); anti‐mono‐ADP‐Ribose (1:1000, HCA354, Bio‐Rad); anti‐Sirt1 (1:1000, AF0282, Beyotime); anti‐Sirt2 (1:1000, D121221, Sangon); anti‐Sirt3 (1:1000, 10099‐1‐AP, Proteintech); anti‐Sirt4 (1:1000, AF7980, Beyotime); anti‐Sirt5 (1:1000, AF2791, Beyotime); anti‐Sirt7 (1:1000, 12994‐1‐AP, Proteintech); anti‐SGCG (1:1000, 18102‐1‐AP, Proteintech); anti‐SNTA1 (1:1000, 13131‐1‐AP, Proteintech); anti‐Flag (1:1000, F1804, Sigma‐Aldrich; 1:1000, 20543‐1‐AP, Proteintech); anti‐Myc (1:1000, sc‐40, Santa Cruz); anti‐HA (1:2000, 51064‐2‐AP, Proteintech); anti‐Histone H3 (1:2000, 17168‐1‐AP, Proteintech); anti‐α‐Tubulin (1:5000, sc‐23948, Santa Cruz); anti‐GAPDH (1:5000, 5174S, Cell Signaling Technology; 1:5000, 60004‐1‐Ig, Proteintech); and anti‐β‐actin (1:3000, 66009‐1‐Ig, Proteintech). HRP‐conjugated goat anti‐mouse and rabbit IgG secondary antibodies were from Thermo (1:5000, 31 430 and 31 460). Immunoreactive bands were visualized using the Super Signal West Femto Maximum Sensitivity Substrate (34 095; Thermo Fisher Scientific) and imaged using the ChemiDoc Touch Imager System (1 708 370; Bio‐Rad Laboratories). Images were analyzed using the ImageJ software.

### 2.6 Histological Analysis

Whole‐mount staining of muscles was carried out as previously described.^[^ ^27^ ^]^ Briefly, tibialis anterior (TA) muscles were fixed in 4% PFA at 4 °C overnight. Subsequently, they were incubated in PBS containing 100 mM glycine for 20 minutes. Muscles were teased into fibers and treated with 1% Triton X‐100 in PBS for 3 hours for permeabilization, and then blocked with a blocking buffer (1% BSA, 0.5% goat serum, 150 mM NaCl, and 0.1% Triton X‐100 in PBS) for 1 hour. The samples were then incubated with the indicated antibodies or R‐BTX. Z‐serial images were captured using a Nikon confocal laser scanning microscope and subsequently analyzed using the ImageJ software.

For hematoxylin and eosin (H&E) staining, TA muscles were excised and rapidly dehydrated using isopentane precooled with liquid nitrogen. Cross‐sections with a thickness of 10 µm were obtained. The samples were stained with hematoxylin and eosin (C0105; Beyotime), and imaged using an upright microscope (Leica DM4000).

### 2.7 Real‐Time PCR

RNA was extracted as previously described.^[^ ^25^ ^]^ Reverse transcription was performed using the iScript cDNA synthesis kit (Bio‐Rad). Real‐time PCR was carried out using the qPCR SYBR Green Master Mix (Yeasen) and GAPDH was used as the reference gene. The primer sequences are indicated in the supplemental information (Table S1).

### 2.8 Chromatin Immunoprecipitation (ChIP)

The ChIP assay was conducted as previously described.^[^ ^28^ ^]^ In brief, C2C12 cells were crosslinked in 1% formaldehyde for 10 minutes, and then quenched by adding 125 mM glycine in PBS. The chromatin was sheared using a sonicator to generate 0.3–0.5 kb DNA fragments, and diluted 10‐fold with IP buffer (50 mM Tris‐HCl, pH 7.6, 150 mM NaCl, 1% NP40, 2.5 mM EDTA, 1 mM PMSF, and protease inhibitor cocktails) to a final concentration of 30 µg/ml DNA. Chromatin lysates were incubated with 2 µg of anti‐YY1 antibody at 4 °C for overnight. Beads were pelleted and successively washed twice with Low Salt buffer (10 mM Tris‐HCl, pH 8.0, 150 mM NaCl, 1 mM EDTA, 1% Triton X‐100, 0.1% SDS), High Salt buffer (10 mM Tris‐HCl, pH 8.0, 500 mM NaCl, 1 mM EDTA, 1% Triton X‐100, 0.1% SDS), LiCl buffer (10 mM Tris‐HCl, pH 8.0, 250 mM LiCl, 1 mM EDTA, 0.5% Na‐deoxycholate, 0.5% NP40) and TE buffer (10 mM Tris‐HCl, pH 8.0, 1 mM EDTA, 50 mM NaCl). DNA was eluted with 200 µl freshly prepared ChIP Elution Buffer (50 mM Tris‐HCl, pH 8.0, 1 mM EDTA, 1% SDS) for 30 min at 65 °C. Reverse crosslinking was conducted in 0.3 M NaCl with water bath at 65° for 16 h, followed by treatment with 0.2 mg/ml RNase (DNase free) at 37 °C for 2 h, and Proteinase K, at 55° for 3 h. The immunoprecipitated genomic DNA was purified using TIANquick Mini Purification Kit (TIANGEN BIOTECH) and resuspended in 20 µl H_2_O. For PCR analysis, 1 µl of the immunoprecipitated DNA was used as a template. The PCR products were analyzed using real‐time PCR on a CFX96 Touch system (Bio‐Rad). The primer information is indicated in Table S1.

### 2.9 Chromatin Binding Assay

Chromatin binding assay was performed as previously described.^[^ ^29^ ^]^ Cells were lysed on ice for 10 minutes using a lysis buffer (50 mM Tris‐HCl, pH 7.6, 150 mM NaCl, 0.1% NP40, 2.5 mM EDTA, 1 mM PMSF, and protease inhibitor cocktails). The lysates were centrifuged at 1000 g for 5 minutes, resulting in supernatant (nonchromatin fraction) and pellet. The pellets were washed three times and dissolved in 0.2 M HCl, followed by sonication. After centrifugation, the supernatants were neutralized with Tris‐HCl (1 M, pH 8.0) and used as the chromatin fraction.

### 2.10 Grip Strength Measurement

Male mice were subjected to grip strength analysis using a grasping force measuring instrument (47 200, Ugo Basile) as previously described.^[^ ^24^ ^‐^ ^26^ ^]^ When the mice grasped a metal grid connected to a force transducer, their tails were gently pulled horizontally to produce a force until the grip was released. Five consecutive trials were performed. Grip strength was standardized to mouse weight in control group of mice as 100%.

### 2.11 Rotarod Test

The rotarod test was conducted as previously described.^[^ ^25^ ^,^ ^26^ ^]^ Male mice were habituated to the rotarod spindle for adapt and trained one day prior to testing. Mice not habituated to the rotarod spindle were excluded from formal rotarod test. During the test, the speed of the rod was 12 rpm per minute. The latency to fall (time) was recorded when the mouse fell off the device. A maximum running time of 12 minutes was set as the cutoff. Five consecutive trials were performed each time.

### 2.12 Electromyography

Electromyography was performed to measure the compound muscle action potential (CMAP) as previously described.^[^ ^27^ ^,^ ^30^ ^,^ ^31^ ^]^ In brief, mice were anesthetized using isoflurane (R510–22, RWD Life Science). A stimulation needle electrode (092‐DMF25‐S, TECA) was inserted near the thigh region. A reference needle electrode was inserted near the Achilles tendon, and a recording needle electrode was inserted into the middle of the gastrocnemius muscle. The reference and recording electrodes were connected to an Axopatch 200B amplifier (Molecular Devices). Supramaximal stimulation was applied to the sciatic nerve using trains of 10 stimuli at frequencies of 1, 5, 10, 20, and 30 Hz (with a 30‐sec pause between each train). CMAPs were collected with a Digidata 1550A (Molecular Devices). The peak‐to‐peak amplitudes of the CAMPs were analyzed using Clampfit10.5 software (Molecular Devices).

### 2.13 Electrophysiological Recording

Electrophysiological recording was conducted as described previously.^[^ ^27^ ^,^ ^30^ ^,^ ^31^ ^]^ In brief, mouse hemidiaphragm with ribs and phrenic nerve distal endings were dissected and pinned on a Sylgard gel substrate in an oxygenated Ringer's solution (136.8 mM NaCl, 5 mM KCl, 12 mM NaHCO_3_, 1 mM NaH_2_PO_4_, 1 mM MgCl_2_, 2 mM CaCl_2_, and 11 mM d‐glucose, pH 7.3) at 26–28 °C. Microelectrodes, filled with 3 M KCl and having a resistance of 20–40 MΩ, were inserted into the myofibers adjacent to the main intramuscular phrenic nerve in the left diaphragm, as visualized under a light microscope. The resting membrane potentials of the recorded muscle fibers remained stable throughout the entire experimental procedure. At least five muscle fibers were recorded from each hemidiaphragm, with a recording duration exceeding 3 minutes. The data were collected using an Axopatch 200B amplifier, digitized (10 kHz low‐pass filtered) with Digidata 1550A, and analyzed using Clampfit 10.5 software.

### 2.14 Proteomics Analysis

To identify differentially expressed proteins in HSA‐Sirt6 cKO mice, label‐free quantitative proteomic analysis were performed in APTBIO, China.^[^ ^32^ ^]^ In brief, TA muscle proteins were extracted using lysis buffer and digested by trypsin and ammonium bicarbonate buffer at 37 °C for 4 hours. Then the samples were incubated with trypsin and CaCl_2_. The supernatant was extracted, filtered, and washed. Liquid chromatography–tandem mass spectrometry (LC‐MS/MS) data collection and database retrieval were performed. The Swissprot_mouse_17 144_20 230 103.fasta database and the MASCOT engine (Matrix Science) embedded in the Proteome Discoverer 1.4 software were utilized to identify the differentially expressed proteins in the samples. The screening of significantly differentially expressed proteins was based on fold change (>1.4 fold for upregulated proteins and <0.714 fold for downregulated proteins) and a p‐value threshold of <0.05. The fold changes of filtered proteins were log2‐transformed and subjected to quantile normalization for generating heat map online (https://www.bioinformatics.com.cn) and volcano plot by GraphPad Prism.

### 2.15 Bioinformatic Analysis

Public microarray dataset was analyzed (GSE55162) to examine expression levels of sirtuin family members in young and old mice. The specific sample information has been described previously.^[^ ^33^ ^]^ In brief, transcript profiling was obtained using distal tracheas and carinas of four young (2 month) and four older (14 month) C57B6 mice. Obtained data were log2‐transformed and subjected to quantile normalization for generating heat map.

### 2.16 Immunoprecipitation–Mass Spectrometry (IP‐Mass)

To identify the YY1‐interacted proteins, C2C12 cells were lysed using a lysis buffer containing 50 mM Tris‐HCl (pH 7.6), 150 mM NaCl, 1% NP40, 2.5 mM EDTA, 1 mM PMSF, and protease inhibitor cocktails. The lysates were centrifuged at 14 000 g for 15 minutes and the supernatants were incubated with anti‐YY1 antibodies and protein A/G agarose beads for overnight at 4 °C. Samples were subjected to SDS‐PAGE and allowed to migrate 1 cm into the separating gel, followed by Coomassie blue staining. The separating gel was cut and subjected to LC‐MS/MS analysis at APTBIO, China.

To detect the mono‐ADP‐ribosylation sites, YY1‐Flag‐transfcted C2C12 cells were lysed and immunoprecipitated using anti‐Flag beads followed by mass spectrometry. During LC‐MS/MS analysis, an aqueous solution of 0.1% formic acid (Liquid A) and an aqueous solution of 0.1% formic acid in acetonitrile (Liquid B) were used. The column was equilibrated with 95% Liquid A, and the sample was loaded onto the Trap column using an autosampler. The mass spectrometry detection mode was positive ion, with a scanning range of 300–1800 m/z for the parent ion. The target value determination was based on Automatic Gain Control (AGC). Dynamic exclusion duration was set at 30.0 ms. Survey scans were acquired at a resolution of 70 000 at m/z 200, and the resolution for HCD spectra was set to 17 500 at m/z 200. The normalized collision energy was 27 eV, and the underfill ratio, which specifies the minimum percentage of the target value likely to be reached at maximum fill time, was defined as 0.1%.

### 2.17 β‐Nicotinamide Mononucleotide (NMN) Treatment

Wild type and HSA‐Sirt6 cKO mice (18‐month‐old) were orally treated daily with either vehicle (H_2_O) or NMN (1.5 mg/ml in H_2_O; BD116593; Bidepharm) for a duration of six months.^[^ ^34^ ^]^ The mice were subjected to behavioral experiments, electrophysiological recording, and biochemical analysis to assess the effects of NMN treatment.

### 2.18 Statistical Analysis

Data were analyzed by the two‐tailed unpaired Student's t test, one‐way ANOVA, or two‐way ANOVA as indicated. The results were presented as mean ± SEM. The GraphPad Prism 7 software was used for statistical analyses and p < 0.05 was accepted as representing statistical significance. *p < 0.05, **p < 0.01, ***p < 0.001, and ****p < 0.0001.

## 3 Results

### 3.1 Sirt6 ablation in Skeletal Muscle Impairs neuromuscular Transmission and Motor Functions

**Figure 1:**
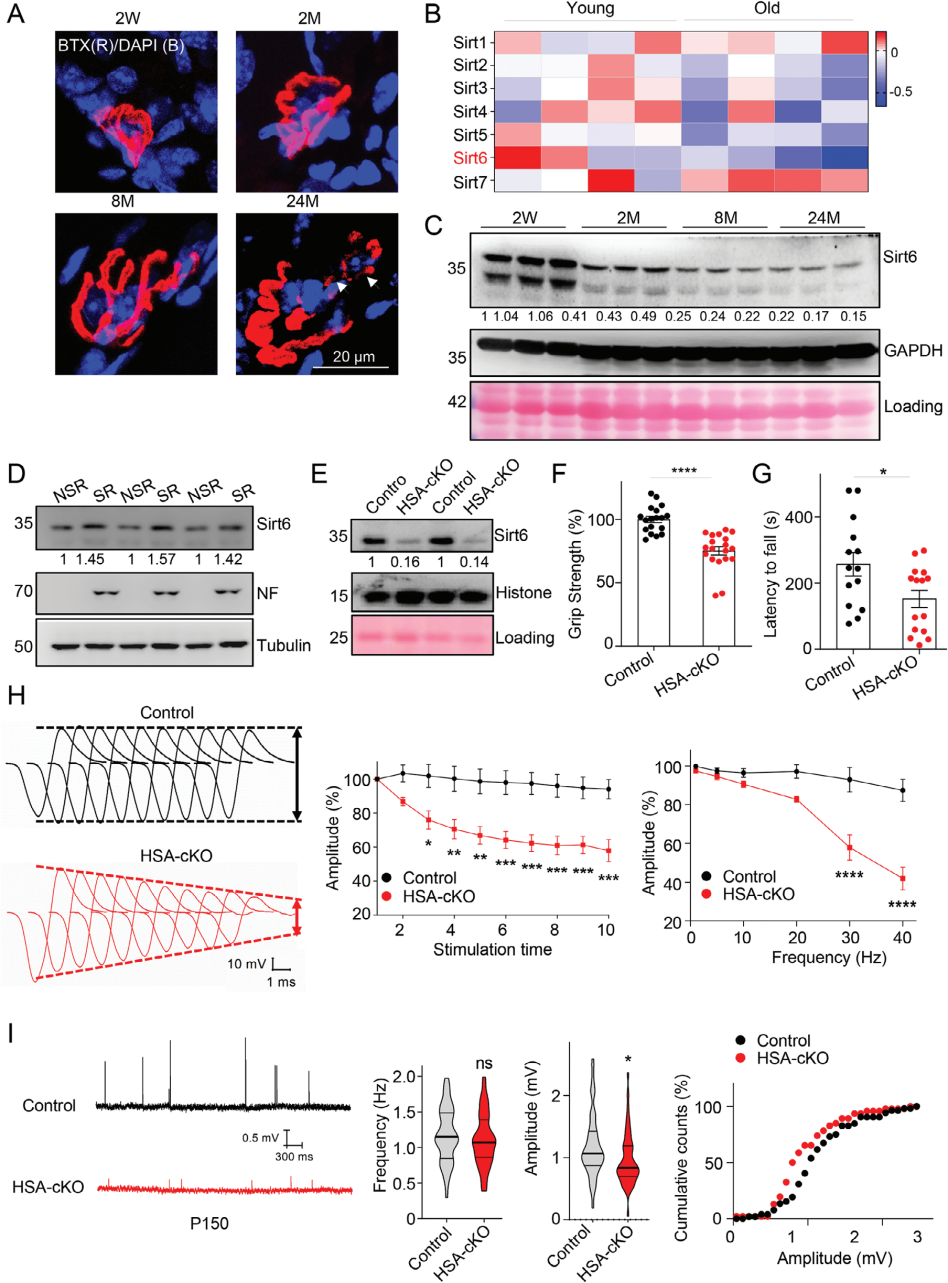
Sirt6 ablation in skeletal muscle impairs neuromuscular transmission and motor functions. (A) Representative fluorescent images show the fragmented NMJs in aged mouse muscles. Tibialis anterior (TA) muscles of indicated ages were stained with R‐BTX (red) and DAPI (blue). Arrows indicate the fragmented NMJs. (B) Heat map of RNA‐seq data shows the expression of Sirtuin family members in young and old mouse trachea samples (NCBI GSE55162). Note that Sirt6 mRNA is specifically reduced in aged samples. (C) Immunoblot shows the reduction of Sirt6 protein levels in aged muscles. TA muscles from mice of different ages were analyzed. W, week; M, month. (D) Enrichment of Sirt6 proteins in the synaptic region (SR) of diaphragm muscles in 4‐month‐old mice. Neurofilament (NF) serves as a positive control for proteins in the synaptic region. Samples were from 3 mice. (E) Immunoblot shows Sirt6 ablation in gastrocnemius muscles in HSA‐Cre; Sirt6^floxp/floxp^ mice (HSA‐Sirt6 cKO, 2‐month‐old). Histone and ponceaus staining indicate loading. (F) Reduced grip strength in HSA‐Sirt6 cKO mice (6‐month‐old). Grip strength was normalized to the body weight. *n* = 18 male mice in control and *n* = 19 male mice in the HSA‐cKO group. (G) Impaired rotarod performance in HSA‐Sirt6 cKO mice (6‐month‐old). *n* = 14 male mice in control and *n* = 15 male mice in the HSA‐cKO group. (H) Impaired neuromuscular transmission in HSA‐Sirt6 cKO mice (5‐month‐old). Left, ten compound muscle action potential (CMAP) traces were stacked in succession for better comparison. CMAPs were recorded in the gastrocnemius in response to a train of 10 submaximal stimuli at different frequencies. CMAP traces between two genotypes at the 1st, 2nd, and 10th stimuli. Middle, CMAP amplitudes at 30 Hz with different stimulation times. Two‐way ANOVA with Sidak's post hoc test for multiple comparisons. Stimulation time: F (9, 60) = 3.934. Amplitude: F (1, 60) = 121.8. Interaction, *p < 0.05; Right, CMAP amplitudes of the tenth stimulation at different stimulation frequencies. *n* = 4 mice in control and *n* = 4 mice in the HSA‐cKO group. Two‐way ANOVA with Sidak's post hoc test for multiple comparisons. Frequency: F (5, 36) = 24.05. Amplitude: F (1, 36) = 63.01. Interaction, ****p < 0.0001. (I) Reduced miniature endplate potential (mEPP) amplitude in HSA‐Sirt6 cKO mice (5‐month‐old). Left: representative mEPP trace; Middle: frequency; Right: amplitude and its cumulative curve. *n* = 53 cells from 6 mice in the control and *n* = 49 cells from 7 mice in the HSA‐Sirt6 cKO group. Unless otherwise specified, at least three independent experiments were performed. Mean ± SEM; *p < 0.05, ***p < 0.001, and ****p < 0.0001; *t*‐test in (F), (G), and (I); two‐way ANOVA in (H).

Neuromuscular junctions (NMJs) exhibit continuous pretzel‐like structure in adult mice. While in aged mouse muscles, NMJs are often fragmented and degenerated (**Figure** **1**A).^[^ ^1^ ^,^ ^2^ ^]^ The underlying mechanism of this degeneration remains unclear. Given the close association of the Sirtuin family with aging, we conducted an analysis of mouse RNA‐seq data from NCBI online database (GSE55162) and found that Sirt6 mRNA levels were specifically reduced in aged samples compared to young samples (Figures 1B). Immunoblot revealed that Sirt6 protein levels in mouse tibialis anterior (TA) muscle were dramatically decreased after 2‐week of age, and continued to diminish gradually during aging (Figures 1C and S1B). Immunofluorescence staining and immunoblot showed that Sirt6 was expressed at a higher level in the synaptic region compared to the non‐synaptic region (Figures S1A and 1D). These findings suggest that Sirt6 may play a role in the structure and/or function of NMJs.

To investigate the role of Sirt6 in muscles, we ablated Sirt6 expression in skeletal muscle by crossing Sirt6‐floxp mice with HSA‐Cre mice (HSA‐Sirt6 cKO, Figure 1E and S1C). The Cre recombinase is specifically expressed in myotomal regions of somites starting from embryonic day 9.5 (E9.5).^[^ ^35^ ^]^ Homozygote HSA‐Sirt6 cKO mice were born in accordance with Mendelian ratios and exhibited normal appearance and body weight during their development (Figure S1D,E). To evaluate the impact of Sirt6 deficiency on motor performance, we conducted grip strength analysis in 6‐month‐old HSA‐Sirt6 cKO mice and revealed a significant reduction compared to the control littermates (Figure 1F). Furthermore, the latency to fall from the rotarod was also reduced in male HSA‐Sirt6 cKO mice (Figure 1G). These results indicate that Sirt6 in skeletal muscle is essential for optimal motor performance.

To investigate whether the motor defects in HSA‐Sirt6 cKO mice are attributed to impaired neuromuscular transmission, we conducted electromyography (EMG) to measure compound muscle action potentials (CMAPs) in response to repetitive nerve stimuli in the sciatic nerve.^[^ ^31^ ^]^ In control littermates, the CMAP amplitude exhibited minimal change after 10 consecutive nerve stimuli at different frequencies (Figure 1H). In contrast, CMAPs in the mutant mice could not be sustained and displayed a frequency‐dependent reduction in the CMAP amplitude (Figure 1H). To ascertain whether the neurotransmission deficits in HSA‐Sirt6 cKO mice are caused by presynaptic and/or postsynaptic impairment, we measured miniature end plate potentials (mEPPs), which are events generated by spontaneous vesicle release. Electrophysiological recordings revealed that mEPP amplitudes were reduced in 5‐month‐old HSA‐Sirt6 cKO mice compared to controls, while the frequency remained unchanged (Figure 1I). These findings suggest that the deletion of Sirt6 primarily impairs postsynaptic assembly at NMJs. Importantly, this effect was not observed in 1.5‐month‐old mice (Figures S1F,G), suggesting that Sirt6 regulates NMJ maintenance rather than development.

### 3.2 Muscle Sirt6 is Essential for Dystrophin Expression and NMJ Maintenance

**Figure 2:**
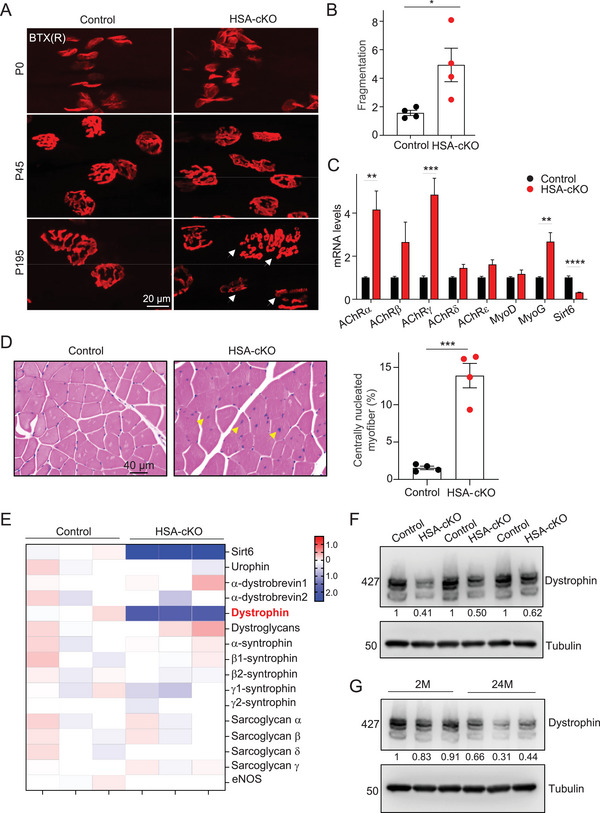
Muscle Sirt6 is required for Dystrophin expression and NMJ maintenance. (A) Representative fluorescent images show fragmented NMJs in diaphragm of HSA‐Sirt6 cKO mice (P195, indicated by white arrows). R‐BTX, red; DAPI, blue. (B) Statistical results of NMJ fragmentation in (A). *n* = 4 mice per group. (C) Real‐time PCR shows aberrant expression of AChR subunits in TA muscles of HSA‐Sirt6 cKO mice (6‐month‐old). (D) H&E staining reveals centralized nucleus in TA muscles of 7‐month‐old HSA‐Sirt6 cKO mice (yellow arrows). Right: quantification results. (E) Heat map of real‐time PCR results shows reduced mRNA levels of Dystrophin in the Dystrophin‐Glycoprotein complex (DGC) in TA muscles of HSA‐Sirt6 cKO mice (6‐month‐old). (F) Immunoblot reveals reduced Dystrophin protein levels in TA muscles of HSA‐Sirt6 cKO mice (6‐month‐old). *n* = 3 mice per group. (G) Immunoblot showing the reduction of Dystrophin protein levels in TA muscles of aged mice (24‐month‐old). *n* = 3 mice per group. Unless otherwise specified, at least three independent experiments were performed. Mean ± SEM; **p < 0.01, ***p < 0.001, and ****p < 0.0001; t‐test in (B‐D).

Subsequently, we employed immunofluorescence staining in HSA‐Sirt6 cKO mice at the neonatal stage (P0) and at one and a half months after birth (P45). We did not observe significant differences in the morphology or size of AChR clusters on the postsynaptic membrane between control and HSA‐Sirt6 cKO muscles (**Figures** **2**A and S2A). However, at a later stage (P195), the NMJ structure exhibited fragmentation in HSA‐Sirt6 cKO mice (Figures 2A,B). Innervating motor neuron terminals were preserved but somewhat distorted in HSA‐Sirt6 cKO muscles (Figure S2B). AChRγ is a subunit of immature AChR complexes, and its expression is upregulated as a compensatory mechanism during NMJ degeneration.^[^ ^36^ ^]^ RT‐PCR analysis revealed an increased mRNA level of AChRγ in TA muscles in HSA‐Sirt6 cKO mice compared to controls (**Figure** 2C). NMJ degeneration is known to cause muscle injury. Indeed, H&E staining unveiled the centralized nuclear localization within some muscle fibers of HSA‐Sirt6 cKO mice (centrally nucleated myofiber: 1.54% in control versus 13.91% in HSA‐Sirt6 cKO mice. Figure 2D). These results collectively suggest that the deficiency of Sirt6 in skeletal muscle leads to NMJ disassembly and premature degeneration.

The Dystrophin‐Glycoprotein complex (DGC) plays a critical role in maintaining AChRs clusters at NMJs.^[^ ^1^ ^,^ ^3^ ^]^ Intriguingly, RT‐PCR analysis revealed that most members of the DGC did not show significant changes in the HSA‐cKO group compared to the control mice (Figure 2E). However, mRNA levels of Dystrophin, the key component of DGC, were notably decreased in the HSA‐Sirt6 cKO group (Figure 2E). Additionally, immunoblot and immunostaining confirmed the reduction of Dystrophin levels in TA muscles of HSA‐Sirt6 cKO mice (Figures 2F, S2C,D), while other DGC components such as Sarcoglycan γ and α1‐Syntrophin showed no significant difference (Figure S2E). Importantly, we observed that Dystrophin mRNA and protein levels were also reduced in aged mice (Figures S2F,G, and 2G). Collectively, these findings indicate that Sirt6 ablation in skeletal muscle leads to decreased Dystrophin expression and impaired NMJ maintenance.

### 3.3 Sirt6 Negatively Regulates the Dystrophin Repressor YY1

**Figure 3:**
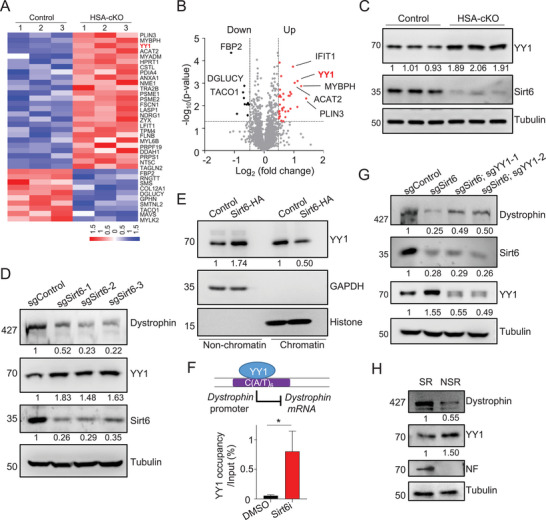
Sirt6 negatively regulates the Dystrophin repressor YY1. (A) Heat map of quantitative proteomic analysis shows differentially expressed proteins in TA muscles of control and HSA‐Sirt6 cKO mice (7‐month‐old). Note that YY1 is upregulated in HSA‐Sirt6 cKO samples. *n* = 3 mice in each group. (B) Volcano plot of quantitative proteomic analysis illustrates upregulated YY1 protein levels in HSA‐Sirt6 cKO mice (7‐month‐old). (C) Immunoblot shows enhanced YY1 protein levels in TA muscles of HSA‐Sirt6 cKO mice (7‐month‐old). (D) Immunoblot shows enhanced YY1 and reduced Dystrophin protein levels in Sirt6 knockout C2C12 cells (sgSirt6). (E) Immunoblot indicate that Sirt6 decreases the chromatin‐bound YY1 levels in C2C12 cells. (F) ChIP assay reveals that Sirt6 negatively regulates the interaction of YY1 with the Dystrophin promoter. The cartoon illustrates YY1 binding to the Dystrophin promoter and repressing its transcription. Bottom: C2C12 cells were treated with the Sirt6 inhibitor OSS‐128167 (Sirt6i, 100 µM, overnight) and subjected for ChIP analysis. (G) Immunoblot shows the requirement of YY1 in Sirt6‐regulated Dystrophin expression. The reduction of Dystrophin levels in Sirt6 knockout C2C12 cells (sgSirt6) was partially rescued in Sirt6 and YY1 double knockout cells (sgSirt6; sgYY1). (H) Immunoblot shows less YY1 and more Dystrophin protein levels in the synaptic region (SR), compared to those in non‐synaptic region (NSR) in the diaphragm of adult mice (6‐month‐old). NF served as a positive control for proteins in the synaptic region. Unless otherwise specified, at least three independent experiments were performed. Mean ± SEM; *p < 0.05; t‐test in (F).

As a histone deacetylase, Sirt6 is known to be involved in the transcriptional repression of genes.^[^ ^8^ ^,^ ^10^ ^]^ In the context of Dystrophin regulation, the downregulation of Dystrophin mRNA and protein in HSA‐Sirt6 cKO mice (Figures 2E,2F) indicates that Sirt6 may inhibit the expression of a negative regulator of Dystrophin, rather than directly regulating Dystrophin itself. To identify potential factors involved, a quantitative proteomics analysis was conducted on TA muscles from control and HSA‐Sirt6 cKO mice. Among the differentially expressed proteins (e.g., IFIT1, YY1, MYBPH, ACAT2, PLIN3 in the upregulated ones; and FBP2, DGLUCY, TACO1 in the downregulated ones), we were particularly interested in YY1 (Ying Yang 1) because it is known as a transcriptional repressor of Dystrophin^[^ ^37^ ^]^ (**Figure** **3**A,B). Immunoblot confirmed the enhancement of YY1 levels in Sirt6‐deficient muscle (Figure 3C). To exclude the possibility of other cell types contributing to the observed effects, we knocked out the Sirt6 expression using CRISPR‐Cas9 in C2C12 muscle cells (Sirt6 KO) and found a reduction of Dystrophin and an increase in YY1 protein levels in Sirt6 KO myotubes (Figure 3D), suggesting that the regulation of YY1 by Sirt6 occurs in a cell‐autonomous manner within muscle cells.

YY1 has been identified as a transcriptional repressor of Dystrophin by binding to the C(A/T)_6_ DNA sequence in the Dystrophin promoter.^[^ ^37^ ^]^ Indeed, chromatin binding assay and chromatin immunoprecipitation (ChIP) analysis revealed that Sirt6 inhibited YY1 binding to the Dystrophin promoter (Figures 3E,F), suggesting that Sirt6 may interfere with YY1‐mediated repression of Dystrophin transcription. To further confirm the involvement of YY1 in the reduction of Dystrophin in Sirt6 KO C2C12 cells, we further generated Sirt6‐YY1 double KO cells and revealed that YY1 ablation partially rescued the reduction of Dystrophin (Figure 3G), providing further evidence for the presence of Sirt6‐YY1‐Dystrophin axis in muscle cells. In addition, Dystrophin was found to be enriched in the synaptic region in mouse diaphragm, while YY1 levels were lower in the synaptic region compared to the non‐synaptic region (Figure 3H). These findings support the notion that Sirt6 negatively regulates the Dystrophin repressor YY1.

### 3.4 Sirt6 Promotes SMURF2 E3 Ligase to Destabilize YY1

**Figure 4:**
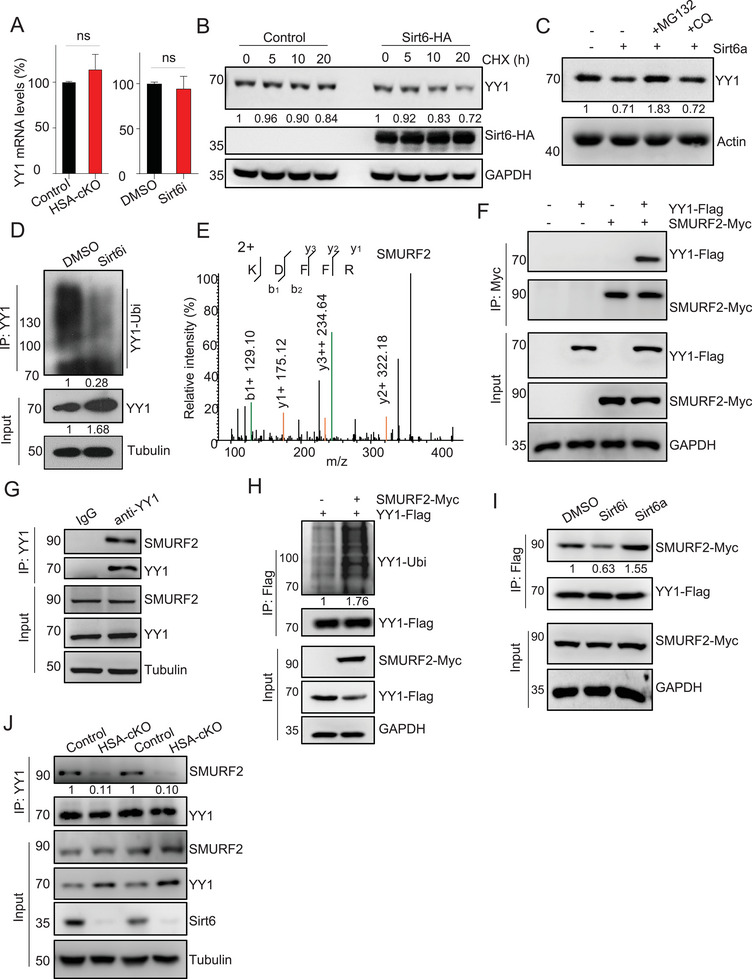
Sirt6 promotes the SMURF2 E3 ligase to destabilize YY1. (A) Real‐time PCR shows no difference of YY1 mRNA levels in 7‐month‐old control and HSA‐cKO TA muscles (left), or vehicle and Sirt6i‐treated C2C12 cells (right). Sirt6i, the Sirt6 inhibitor OSS‐128167. (B) Chase analysis shows the shorter half‐life of YY1 proteins in Sirt6‐expressed C2C12 cells. Cells were treated with cycloheximide (CHX, 10 µM) for the indicated hours before harvesting for immunoblot. (C) Immunoblot indicates that Sirt6‐mediated YY1 reduction depends on the ubiquitination‐proteasome system rather than the autophagy‐lysosome pathway. C2C12 cells were pretreated with Sirt6a (10 µM, overnight), followed by treatment of MG132 (10 µM, 2 hours) or Chloroquine (CQ, 10 µM, 2 hours) before harvesting.^[^ ^23^ ^,^ ^26^ ^]^ Sirt6a, the Sirt6 agonist MDL‐800; MG132, the inhibitor of ubiquitination‐proteasome system; CQ, the inhibitor of autophagy‐lysosome pathway. (D) Immunoprecipitation and immunoblot show that the Sirt6 inhibitor reduces the ubiquitination of YY1 in C2C12 cells. (E) Peptide information of SMURF2 E3 ligase in the proteomics result. C2C12 cells were lysed and subjected to IP‐MASS (anti‐YY1 antibodies) to identify its interacting proteins. (F) Co‐immunoprecipitation shows the interaction between YY1‐Flag and SMURF2‐Myc proteins in C2C12 cells. (G) Co‐immunoprecipitation shows the interaction between endogenous YY1 and SMURF2 proteins in adult mouse TA muscles. (H) Immunoprecipitation and immunoblot show that SMURF2 promotes YY1 ubiquitination in C2C12 cells. (I) Immunoblot indicates that Sirt6 activity regulates the interaction between YY1 and SMURF2 in C2C12 cells. (J) Co‐immunoprecipitation shows that Sirt6 is required for the interaction between YY1 and SMURF2 in mouse TA muscles (7‐month‐old). Unless otherwise specified, at least three independent experiments were performed. Mean ± SEM; t‐test in (A).

We next sought to determine whether Sirt6 negatively regulates YY1 expression by inhibiting its transcription. Surprisingly, RT‐PCR did not show an increase in YY1 mRNA levels in HSA‐Sirt6 cKO muscles or in Sirt6 inhibitor‐treated C2C12 cells (**Figure** **4**A). This suggests that Sirt6 may regulate YY1 at the protein level rather than affecting its transcription. A chase experiment showed that the half‐life of YY1 proteins was reduced in C2C12 cells expressing Sirt6 (Figure 4B). This effect appeared to be dependent on ubiquitination‐proteasome degradation, as the Sirt6‐mediated reduction of YY1 was blocked by MG132 (a proteasome inhibitor) but not by Chloroquine (CQ, an autophagy inhibitor) treatment (Figure 4C). Additionally, a significant decrease in the ubiquitination level of YY1 was observed in C2C12 cells treated with the Sirt6 inhibitor (Figure 4D). These findings indicate that Sirt6 regulates the ubiquitination of YY1, leading to its proteasomal degradation.

As a potent regulator in gene expression, the YY1 protein is ubiquitously expressed, and its protein levels are tightly controlled by different E3 ligases in various cell types.^[^ ^38^ ^‐^ ^42^ ^]^ To identify the potential E3 ligase(s) involved in the Sirt6‐mediated ubiquitination of YY1 in muscles, we performed IP‐Mass and found the presence of the E3 ligase SMURF2 in the list of identified proteins (Figure 4E). Co‐IP assay confirmed the exogenous and endogenous interactions between SMURF2 and YY1 (Figure 4F,G). Furthermore, SMURF2 promoted the ubiquitination of YY1, leading to the reduction of YY1 protein levels (Figure 4H). Interestingly, we found that Sirt6 regulated the interaction of SMURF2 and YY1 in C2C12 cells and mouse TA muscles (Figures 4I,J). Taken together, these results strongly suggest that Sirt6 promotes the SMURF2 E3 ligase to destabilize YY1 through ubiquitination and subsequent degradation.

### 3.5 Sirt6 Mono‐ADP‐Ribosylates YY1 to Release it from the Dystrophin Promoter for Degradation

**Figure 5:**
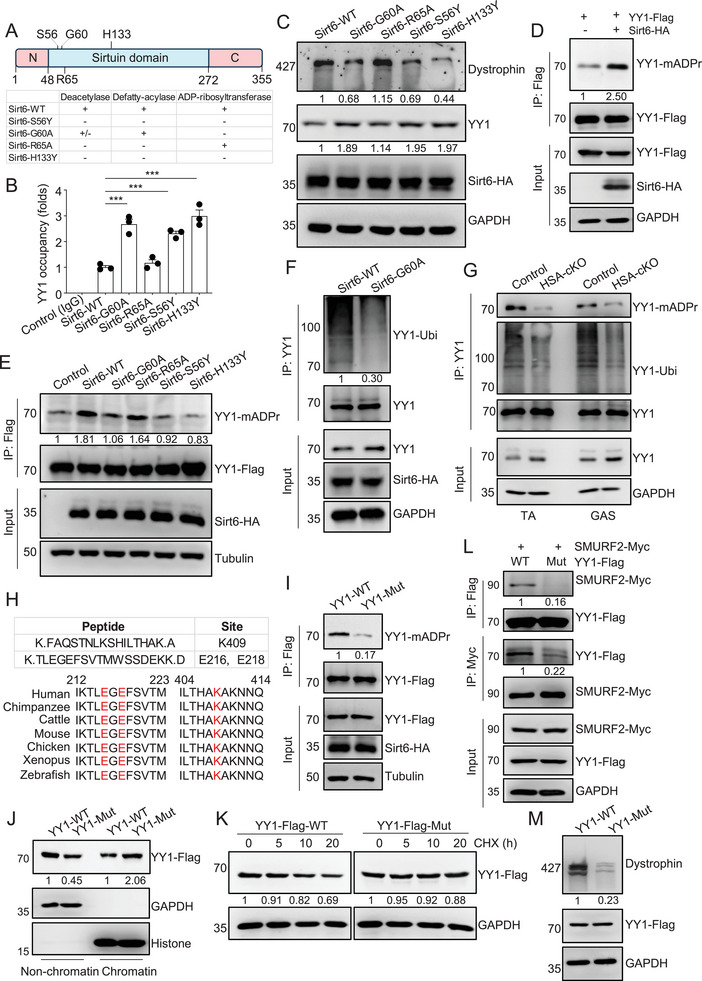
Sirt6 mono‐ADP‐ribosylates YY1 to release it from the Dystrophin promoter for degradation. (A) The cartoon and table illustrate the defects in enzyme activities of Sirt6 mutants. (B) ChIP assay shows that the activity of Sirt6 mono‐ADP‐ribosyltransferase inhibits its binding on the Dystrophin promoter. C2C12 cells were transfected with different Sirt6‐HA mutants and subjected to ChIP analysis using anti‐YY1 antibodies. WT, wild type. One‐way ANOVA with Tukey's multiple comparisons test. F (5, 12) = 64.30. (C) Immunoblot shows that the activity of Sirt6 mono‐ADP‐ribosyltransferase is required for Dystrophin expression in C2C12 cells. (D) Immunoprecipitation and immunoblot show that Sirt6 promotes the mono‐ADP‐ribosylation of YY1 in C2C12 cells. (E) Immunoprecipitation and immunoblot show that Sirt6‐G60A cannot mono‐ADP‐ribosylate YY1 in C2C12 cells. (F) Immunoblot indicates that the activity of Sirt6 mono‐ADP‐ribosyltransferase is required for YY1 ubiquitination. (G) Immunoblot shows the reduction of YY1 mono‐ADP‐ribosylation and ubiquitination in TA muscles of HSA‐Sirt6 cKO mice (7‐month‐old). (H) Peptide information of YY1 mono‐ADP‐ribosylation sites in the proteomics result (top). The consensus of YY1 mono‐ADP‐ribosylation sites (E206/E208/K409 in the human sequence) is indicated in red (bottom). YY1‐Flag‐tansfected C2C12 cells were lysed and subjected to IP‐MASS (anti‐Flag antibodies) to identify its modification sites. (I) Immunoblot shows a dramatic reduction in mono‐ADP‐ribosylation in the YY1‐E206A/E208A/K409A mutant. C2C12 cells were transfected with Sirt6‐HA and YY1‐Flag (WT or mutant), and their lysates were subjected to detection of YY1 mono‐ADP‐ribosylation. (J) Immunoblot shows an enhanced interaction between YY1 with chromatin when the mono‐ADP‐ribosylation sites in YY1 were mutated. C2C12 cells were transfected with YY1‐Flag (WT or mutant), and their lysates were separated into chromatin and non‐chromatin fractions before immunoblot. (K) Chase analysis reveals a longer half‐life of YY1 proteins when the mono‐ADP‐ribosylation sites in YY1 were mutated. C2C12 cells were transfected with YY1‐Flag (WT or mutant) and treated with CHX (10 µM) for the indicated hours before harvesting for immunoblot. (L) Immunoprecipitation and immunoblot show that mono‐ADP‐ribosylation of YY1 is necessary for the interaction between YY1 and SMURF2 proteins. (M) Immunoblot shows that mono‐ADP‐ribosylation of YY1 is required for Dystrophin expression. C2C12 cells were transfected with YY1‐Flag (WT or mutant) and harvested for immunoblot. Unless otherwise specified, at least three independent experiments were performed. Mean ± SEM; one‐way ANOVA in (B).

Sirt6 possesses deacetylation, defatty‐acylation, and mono‐ADP‐ribosylation activities. Previous studies have identified that the amino acid R65 is critical for deacetylation activity, G60 is critical for mono‐ADP‐ribosylation activity, and S56 and H133 are critical for both activity^[^ ^10^ ^,^ ^43^ ^]^ (**Figure** **5**A). To determine which enzymatic activity of Sirt6 is critical for regulating YY1 stability, different Sirt6 mutants were transfected into C2C12 cells. Intriguingly, we found that Sirt6‐G60A, Sirt6‐S56Y, and Sirt6‐H133Y mutants, which disrupted the mono‐ADP‐ribosylation activity of Sirt6, all exhibited increased binding of YY1 to the Dystrophin promoter compared to the wild‐type (WT) control and Sirt6‐R65A (Figure 5B). This suggests that the mono‐ADP‐ribosylation activity of Sirt6 inhibits YY1 from binding to the Dystrophin promoter as a transcription repressor. Consistently, Dystrophin protein levels were reduced when the sites for mono‐ADP‐ribosylation activities were mutated in the Sirt6‐G60A, Sirt6‐S56Y, and Sirt6‐H133Y mutants (Figure 5C), further supporting the requirement of the mono‐ADP‐ribosylation activity of Sirt6 for the regulation of Dystrophin expression.

We next investigated whether Sirt6 is capable of mono‐ADP‐ribosylating YY1. Indeed, we found that Sirt6‐WT enhanced the levels of mono‐ADP‐ribosylated YY1 (YY1‐mADPr), whereas the Sirt6‐G60A mutation abolished this effect (Figures 5D,E). Furthermore, the ubiquitination of YY1 decreased in Sirt6‐G60A samples and HSA‐Sirt6 cKO muscles (Figures 5F,G, and S4A), suggesting that mono‐ADP‐ribosylation of YY1 promotes its ubiquitination and subsequent destabilization.

To pinpoint the specific site(s) in YY1 undergoing mono‐ADP‐ribosylation by Sirt6, we immunoprecipitated YY1 and subjected to proteomic analysis of its mono‐ADP‐ribosylation modifications. Three potential sites, E216, E218, and K409, were identified in the proteomic analysis (Figures 5H and S3). We mutated all these three sites (YY1‐E216A/E218A/K409A) and found that Sirt6‐mediated mono‐ADP‐ribosylation was abolished in the mutant (Figure 5I). Interestingly, the YY1‐E216A/E218A/K409A mutant increased its chromatin binding, suggesting that mono‐ADP‐ribosylation inhibits YY1 from binding to chromatin. Furthermore, the YY1‐mutant failed to interact with the SMURF2 E3 ligase and displayed enhanced stability compared to YY1‐WT (Figures 5J‐5L), resulting in the inhibition of Dystrophion expression (Figure 5M). Together, these findings suggest that Sirt6 mono‐ADP‐ribosylates YY1, leading to its release from the Dystrophin promoter, thereby promoting its degradation.

### 3.6 NMN Increases Mono‐ADP‐Ribosylation of YY1 and Alleviates Motor Defects in Aged Muscles

**Figure 6:**
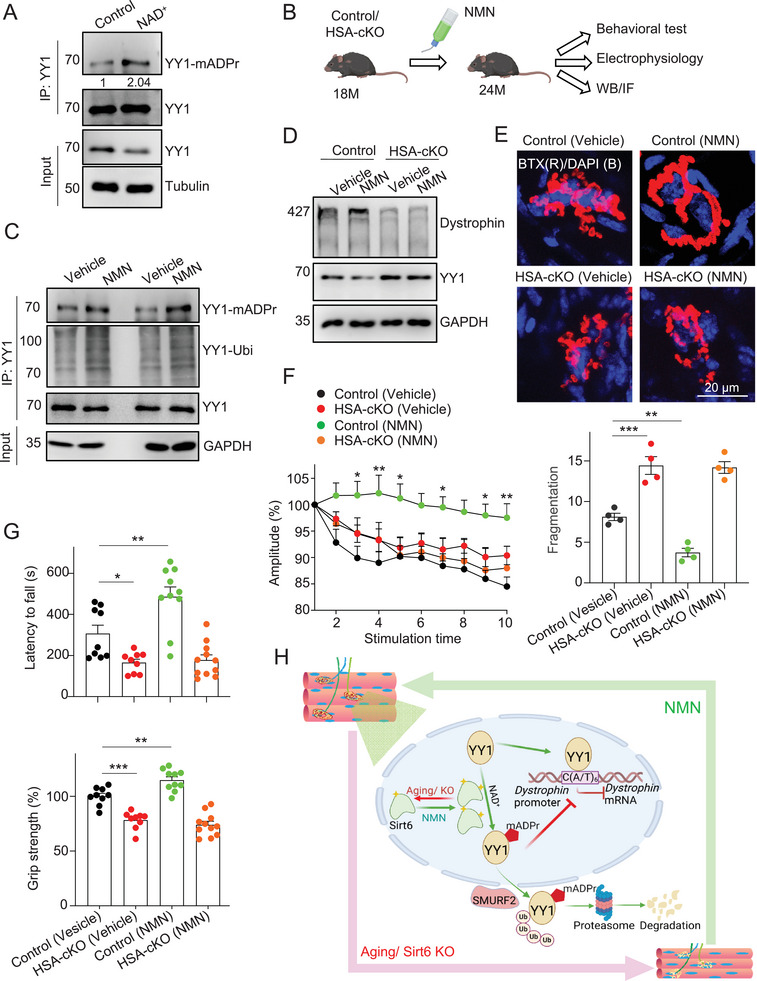
NMN (β‐Nicotinamide mononucleotide) treatment increases mono‐ADP‐ribosylation of YY1 and alleviates motor defects in aged mice. (A) Immunoprecipitation and immunoblot show that NAD^+^ promotes mono‐ADP‐ribosylation of YY1. C2C12 cells were treated with NAD^+^ (100 µM, overnight)^[^ ^64^ ^]^ and harvested for mono‐ADP‐ribosylation of YY1 analysis. (B) The experiment procedure of NMN treatment. 18‐month‐old WT or HSA‐Sirt6 cKO mice were orally administered with NMN (1.5 mg/ml in H_2_O) for 6 months before behavior analysis, electrophysiology, and immunoblot (Created in BioRender. Zhang, Z. (2020) BioRender.com/j41q476). (C) Immunoprecipitation and immunoblot show that NMN treatment enhances mono‐ADP‐ribosylation and ubiquitination of YY1 in TA muscles of aged mice (24‐month‐old). (D) Immunoblot shows that NMN treatment increases the levels of Dystrophin protein in TA muscles of aged WT mice (24‐month‐old). It is noteworthy that NMN has little effect in aged HSA‐Sirt6 cKO mice. (E) Representative fluorescent images show that NMN treatment reduces NMJ fragmentation in aged mouse muscles (24‐month‐old, above). TA muscles were stained with R‐BTX (red) and DAPI (blue). Statistical results (below). *n* = 4 mice per group. One‐way ANOVA with Tukey's multiple comparisons test. F (3, 12) = 48.90. (F) Electromyography analysis shows that NMN treatment increases the reduction of CMAPs amplitude in aged WT mice (24‐month‐old). WT (vehicle), *n* = 5 mice; WT (NMN treatment), *n* = 5 mice; HSA‐Sirt6 cKO (vehicle), *n* = 5 mice; HSA‐Sirt6 cKO (NMN treatment), *n* = 4 mice. Two‐way ANOVA with Sidak's post hoc test for multiple comparisons. Stimulation time: F (9, 150) = 6.702. Amplitude: F (3, 150) = 36.93. (G) Improved motor performance in NMN‐treated aged WT mice (24‐month‐old). Above: rotarod performance; Below: grip strength. WT (Vehicle), *n* = 9 mice; WT (NMN treatment), *n* = 10 mice; HSA‐Sirt6 cKO (Vehicle), *n* = 9 mice; HSA‐Sirt6 cKO (NMN treatment), *n* = 11 mice. One‐way ANOVA with Tukey's multiple comparisons test. Rotarod: F (3, 35) = 18.70, Grip strength: F (3, 35) = 43.32. (H) The working model (Created in BioRender. Zhang, Z. (2021) BioRender.com/f79z500). Unless otherwise specified, at least three independent experiments were performed. Mean ± SEM; *p < 0.05, **p < 0.01, and ***p < 0.001; one‐way ANOVA in (E) and (G); two‐way ANOVA in (F).

Sirt6 activity is known to be NAD^+^‐dependent. Indeed, we found that NAD^+^ treatment enhanced mono‐ADP‐ribosylation of YY1 (**Figure** **6**A). We next asked whether supplement of NAD^+^ could alleviate NMJ degeneration and motor defects in aged mice. Due to its large molecular size, NAD^+^ itself is difficult to be absorbed by human body. As a precursor of NAD^+^, NMN is relatively small and can be rapidly absorbed by the intestinal epithelium and converted to NAD^+^ intracellularly.^[^ ^44^ ^,^ ^45^ ^]^ While its effects in human studies are still debated, NMN has been reported to have beneficial effects on aged animals.^[^ ^11^ ^,^ ^45^ ^,^ ^46^ ^]^ The impact of NMN on NMJ degeneration in aged mouse muscles remains unknown. To address this, we fed the old mice (18‐month‐old) with NMN for 6 months and assessed their motor behaviors and neurotransmission (Figure 6B).

We found that NMN treatment increased the levels of mono‐ADP‐ribosylation and ubiquitination of YY1. Importantly, Dystrophin protein levels were enhanced in the aged mice treated with NMN. However, this effect was not observed in old HSA‐Sirt6 cKO mice, suggesting that the impact of NMN on YY1 mono‐ADP‐ribosylation and Dystrophin expression was primarily mediated through Sirt6 but not other proteins (Figure 6C,D, S4B,C). Importantly, we observed that NMN treatment reduced NMJ degeneration (Figures 6E). In line with these findings, NMN‐treated control mice exhibited improvements in CMAP amplitudes and motor behaviors, including grip strength and rotarod performance. These effects were not observed in HSA‐Sirt6 cKO mice (Figures 6F,G). Together, these results showed that NMN plays a beneficial role in alleviating motor defects in aged mice by increasing the mono‐ADP‐ribosylation of YY1, thereby promoting Dystrophin expression.

## 4 Discussion

Aging is associated with NMJ degeneration and a decline in motor functions, yet the underlying mechanisms remain poorly understood. Our study revealed that Sirt6 in skeletal muscles alleviates the inhibitory effect of YY1 on Dystrophin expression for NMJ maintenance. Boosting Sirt6 activity through NMN supplement can delay NMJ degeneration and improve motor functions in aged mice.

Sirt6 is recognized to be involved in various physiological and pathological processes. Animals lacking Sirt6 display premature degeneration in multiple organs, characterized by reduced bone density, lymphocyte loss, decreased subcutaneous fat, and muscle atrophy, leading to a shorter lifespan.^[^ ^8^ ^,^ ^12^ ^,^ ^13^ ^]^ However, the mechanism by which Sirt6 deficiency contributes to age‐related decline in motor functions remain elusive. We observed that Sirt6 deficiency in skeletal muscles leads to premature degeneration of NMJ structure and impaired neuromuscular transmission, indicating that Sirt6 is crucial for the maintenance of NMJ structure and function. Dystrophin, a protein found along muscle fibers, is expressed at higher levels in the synaptic region of NMJ compared to the non‐synaptic region (Figure 3).^[^ ^47^ ^,^ ^48^ ^]^ The Agrin‐Lrp4‐MuSK‐Dok7‐Rapsyn‐AChR pathway is critical for NMJ formation and maintenance.^[^ ^1^ ^]^ There are two possible mechanisms to link our findings to the Agrin signaling in NMJ maintenance. 1) Agrin, a nerve‐derived extracellular matrix protein, binds to the DGC component α‐Dystroglycan.^[^ ^49^ ^‐^ ^52^ ^]^ This extracellular interaction may locally anchor Agrin at the synaptic region with high density for NMJ maintenance. In Sirt6 null muscle, the loss of Dystrophin may cause DGC disassembly, ultimately leading to a less availability of Agrin at the synapse. 2) The intracellular scaffold protein Rapsyn not only clusters AChR, but also β‐Dystroglycan.^[^ ^53^ ^,^ ^54^ ^]^ The clustered DGC‐Rapsyn‐AChR may provide a cytoskeletal anchor necessary for NMJ stabilization. The loss of Dystrophin in Sirt6 null muscle may cause DGC disassembly, eventually leading to a reduced association with Rapsyn and less stabilization of AChR clusters. Sirt6 and dystrophin are known to play roles in autophagy and muscle satellite cell functions.^[^ ^55^ ^‐^ ^57^ ^]^ Impaired autophagy contributes to declined muscle regeneration during aging.^[^ ^58^ ^,^ ^59^ ^]^ We find that the Sirt6 is required to prevent NMJ degeneration through regulating dystrophin level. This could also explain the reduced muscle regeneration and impaired motor functions in aging.

Sirt6 possesses NAD^+^‐dependent deacetylase, mono‐ADP‐ribosyltransferase, and limited deacylase activity.^[^ ^8^ ^]^ While most research on Sirt6 in the nucleus has focused on its role as a Histone deacetylase in regulating gene expression, the understanding of its ADP‐ribosyltransferase activity is not well understood. In our study, we unexpectedly found that Sirt6 regulates YY1 expression at its protein level, but not at the mRNA level. This regulation is mediated by the mono‐ADP‐ribosyltransferase activity of Sirt6, rather than its deacetylase or deacylase activity (Figure 5). The mono‐ADP‐ribosylation of YY1 leads to a decrease in its ability to bind to the Dystrophin promoter, possibly due to the addition of negative charge to YY1. Consequently, free YY1 is targeted by the E3 ligase SMURF2 and undergoes ubiquitination, leading to its proteasomal degradation (Figure 6, the working model). Although Sirt6 is associated with YY1, the regulation between these two molecules was unclear.^[^ ^60^ ^,^ ^61^ ^]^ Our findings provide the first evidence that Sirt6 is the critical enzyme for the mono‐ADP‐ribosylation of YY1, expanding our knowledge of the diverse functions of Sirt6 beyond its well‐known deacetylase activity. Sirt6 was reported to deacetylate H3K56ac to suppress utrophin expression in dystrophin‐deficient mdx mice.^[^ ^19^ ^]^ The mechanisms underlying the transition of enzyme activities of Sirt6 between physiological and pathological situations could be a charming question. As a powerful regulator of gene expression, YY1 undergoes multiple post‐translational modifications. Our study reveals an intriguing interplay between ubiquitination and mono‐ADP‐ribosylation in modulating the function of YY1.

The enzymatic activity of Sirt6 is dependent on NAD^+^ levels, but NAD^+^ has difficulty in being absorbed by the human body due to its large molecular size. As a precursor to NAD^+^, NMN availability is critical for mammalian NAD^+^ biosynthesis. NMN is relatively small and can be rapidly absorbed by the intestinal epithelium. It serves as an intermediate metabolite in certain natural foods and is considered a safe health supplement with no significant reported side effects.^[^ ^11^ ^]^ Studies have indicated that NMN possesses anti‐aging properties, including the improvement of human motor function.^[^ ^11^ ^]^ However, the downstream mechanisms through which NMN exerts its effects are not yet fully understood, given the broad impact of NAD^+^ on various cellular reactions. Our findings have provided valuable insights by demonstrating that NMN improves NMJ functions in aged WT mice but not in Sirt6‐deficient mice, suggesting that the primarily action of NMN in improving age‐related motor function is mediated through the Sirt6 protein. Consistently, overexpressing Sirt6 in transgenic mice was reported to improve motor behaviors and life span.^[^ ^14^ ^,^ ^20^ ^]^ Although the dose of NMN in our mouse study appears to be theoretically tolerable in humans,^[^ ^62^ ^,^ ^63^ ^]^ further rigorous exploration is necessary to determine whether this dosage is truly physiologically suitable for human use.

In conclusion, our research significantly contributes to our understanding of the structural and functional degeneration of NMJ in aged muscles. Targeting Sirt6 could be a promising therapeutic strategy to combat the decline in motor functions associated with aging.

## Conflict of Interest

The authors declare no conflict of interest.

## Author Contributions

C.S., K.Z., and G.P.: conceptualization, supervision, resources, funding, and writing. W.Z., L.B., and W.X.: methodology, investigation, and writing. J.L., Y.C., W.L., H.L., B.W., and B.L.: Investigation.

## Supporting information



Supporting Information

## Data Availability

The data that support the findings of this study are available from the corresponding author upon reasonable request.
