# Supplementary material for: Sirt6 Mono‐ADP‐Ribosylates YY1 to Promote Dystrophin Expression for Neuromuscular Transmission
Source: Adv Sci (Weinh). 2024 Oct 10;11(44):2406390. doi: 10.1002/advs.202406390 (PMC11600243; doi:10.1002/advs.202406390)
Supplement: Supplementary file 1 — Supporting Information [file ADVS-11-2406390-s001.docx]

Supporting Information

**Sirt6 Mono-ADP-ribosylates YY1 to Promote Dystrophin Expression for Neuromuscular Transmission**

Wei Zhang^1,8^, Lei Bai^1,8^, Wentao Xu^1,8^, Jun Liu^2^, Yi Chen^5^, Weiqiang Lin^3^, Huasong Lu^4^, Binwei Wang^2^, Benyan Luo^5^, Guoping Peng^5,9^, Kejing Zhang^6,9^, Chengyong Shen^1,7,9^

Correspondence:

Guoping Peng, guopingpeng@zju.edu.cn

Kejing Zhang, kjzhang@zju.edu.cn

Chengyong Shen, [cshen@zju.edu.cn](mailto:cshen@zju.edu.cn)


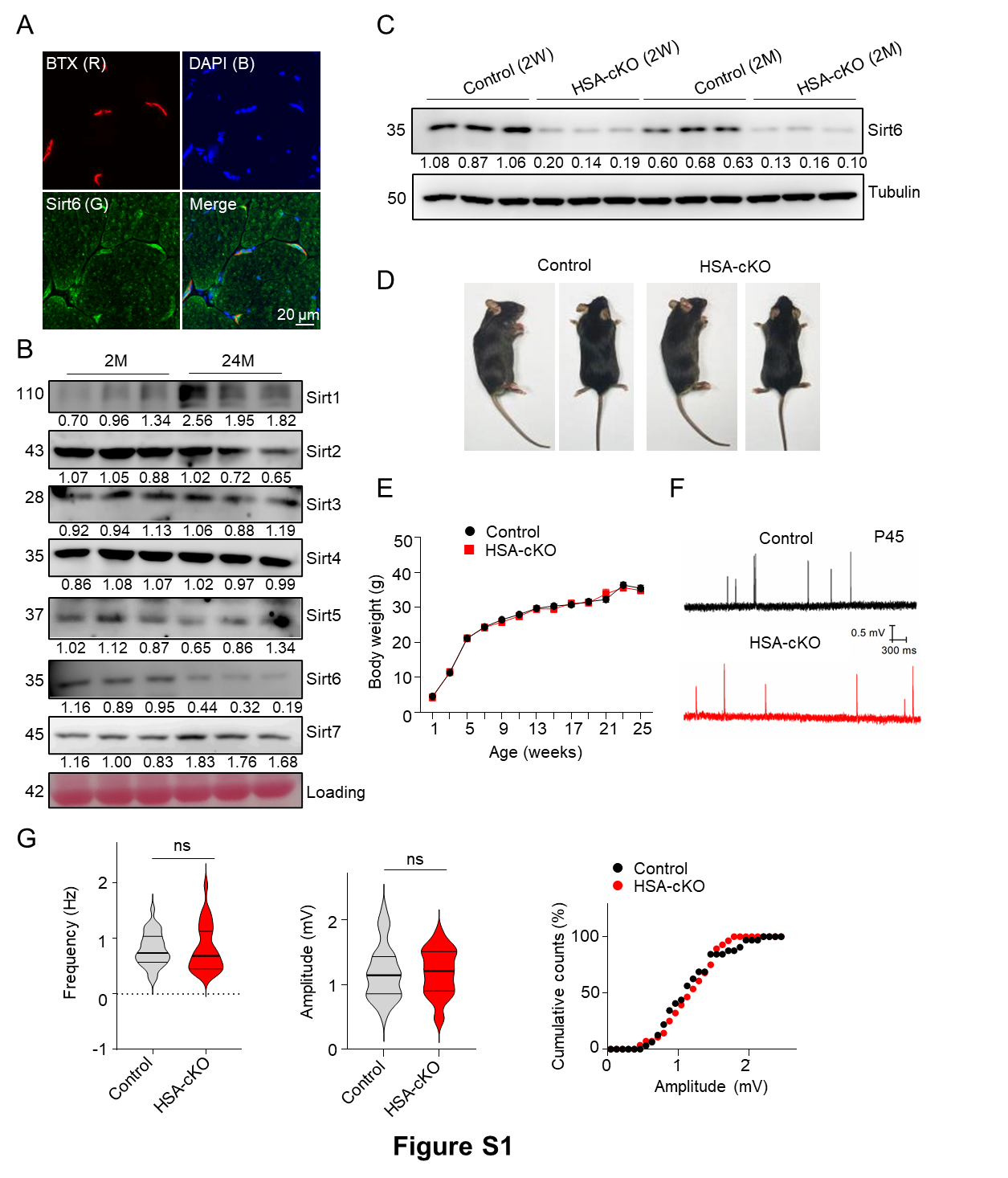


**Figure S1. Sirt6 functions in neuromuscular synapse (relates to Figures 1 and 2).**

1. Representative fluorescent images show the presence of Sirt6 at NMJs in cross section of gastrocnemius muscles in adult mice. Sirt6, green; BTX, red; DAPI, blue.
2. Immunoblot of Sirt1-7 proteins in skeletal muscles of young and aged mice. Gastrocnemius muscles were from 2-month-old and 24 month-old mice. n = 3 mice per group.
3. Immunoblot shows Sirt6 ablation in gastrocnemius muscles in HSA-Sirt6 cKO mice at indicated ages.
4. Normal body size of HSA-Sirt6 cKO mice (2-month-old).
5. Normal body weight of HSA-Sirt6 cKO male mice at indicated ages.
6. Representative mEPP trace in control and HSA-Sirt6 cKO mice at 1.5-month-old mice.
7. Left: mEPP frequency; Middle: mEPP amplitude; Right: cumulative curve of mEPP amplitude. n = 32 cells from 4 mice in the control and n = 28 cells from 4 mice in the HSA-Sirt6 cKO group.

Unless otherwise specified, at least three independent experiments were performed. Mean ± SEM; one-way ANOVA in (E); t-test in (G).


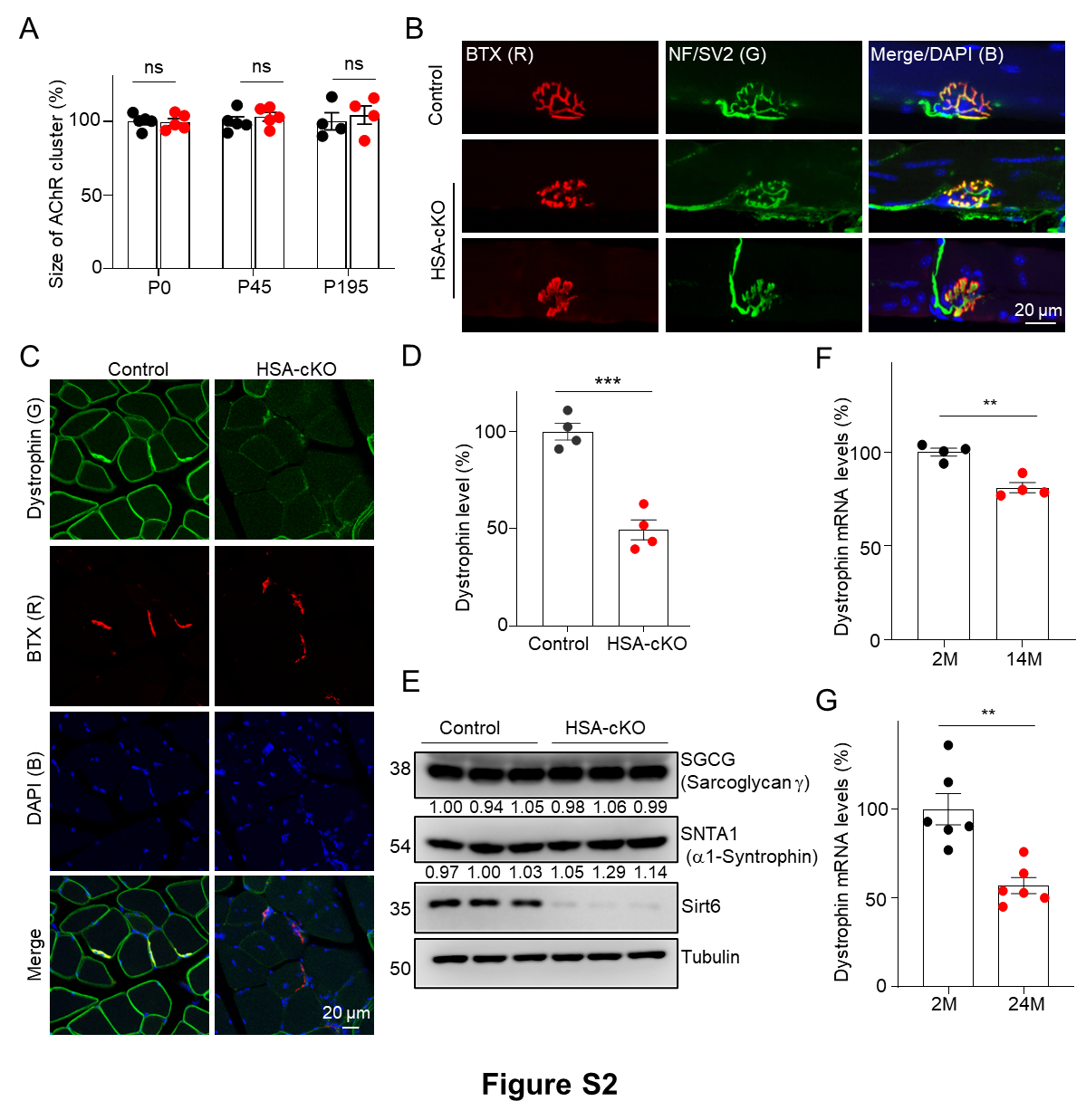


**Figure S2. Muscle Sirt6 regulates Dystrophin expression and NMJ maintenance. (relates to Figure 2).**

1. Normal NMJ size in HSA-Sirt6 cKO mice at indicated ages.
2. Representative fluorescent images show the innervation of NMJ in control and HSA-Sirt6 cKO mouse. TA muscles from 6-month-old mice were analyzed. Anti-Neurofilament and SV2, green; BTX, red; DAPI, blue.
3. Representative fluorescent images show the reduction of Dystrophin in HSA-Sirt6 cKO muscles. Gastrocnemius muscles were from 8-month-old mice.
4. Statistical results of (C).
5. Immunoblot shows little difference of Sarcoglycan γ and α1-Syntrophin in TA muscles of HSA-Sirt6 cKO mice (6-month-old). n = 3 mice per group.

(F and G) Dystrophin mRNA levels in mouse samples at indicated ages. Data in (C) were from database NCBI GSE55162, and data in (D) were from mouse TA muscles

Unless otherwise specified, at least three independent experiments were performed. Mean ± SEM; **p < 0.01; ***p < 0.001; t-test in (A), (D), (F), and (G).


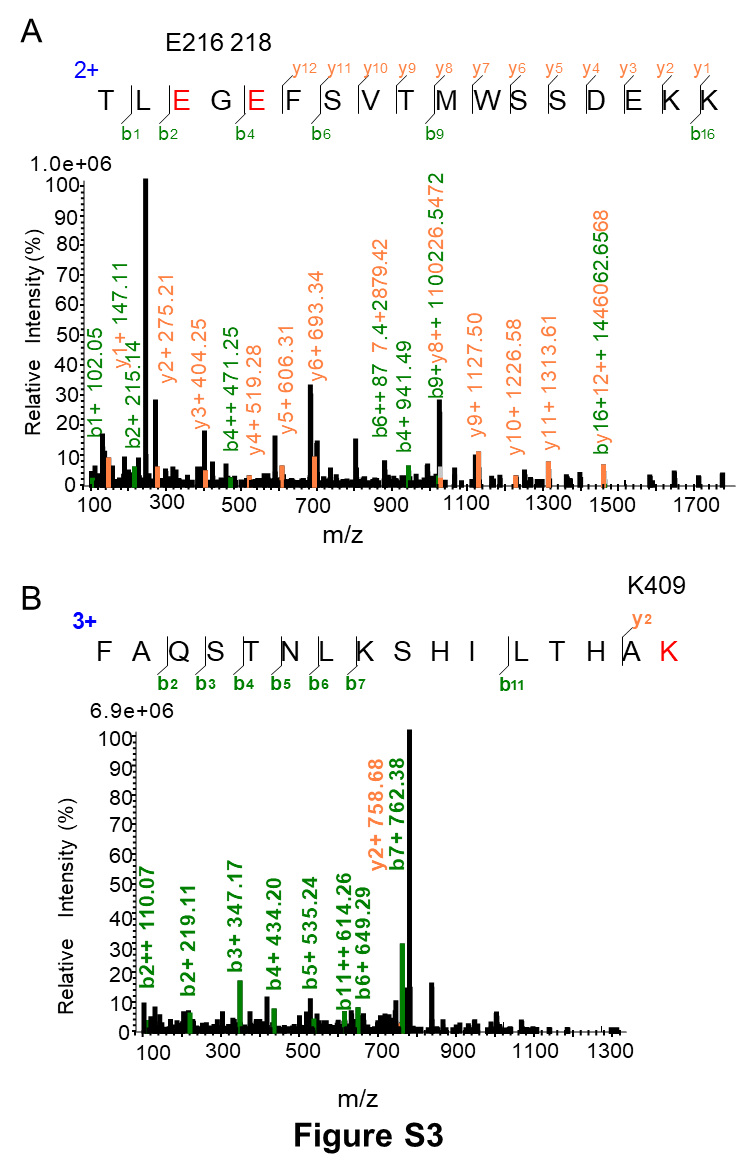


**Figure S3. Identification of mono-ADP-ribosylation sites in YY1 (relates to Figure 5).**

(A and B). Peptide information of YY1 mono-ADP-ribosylation sites in the proteomics result. YY1-Flag-tansfected C2C12 cells were lysed and subjected to IP-MASS (anti-Flag antibodies) to identify its modification sites.

**
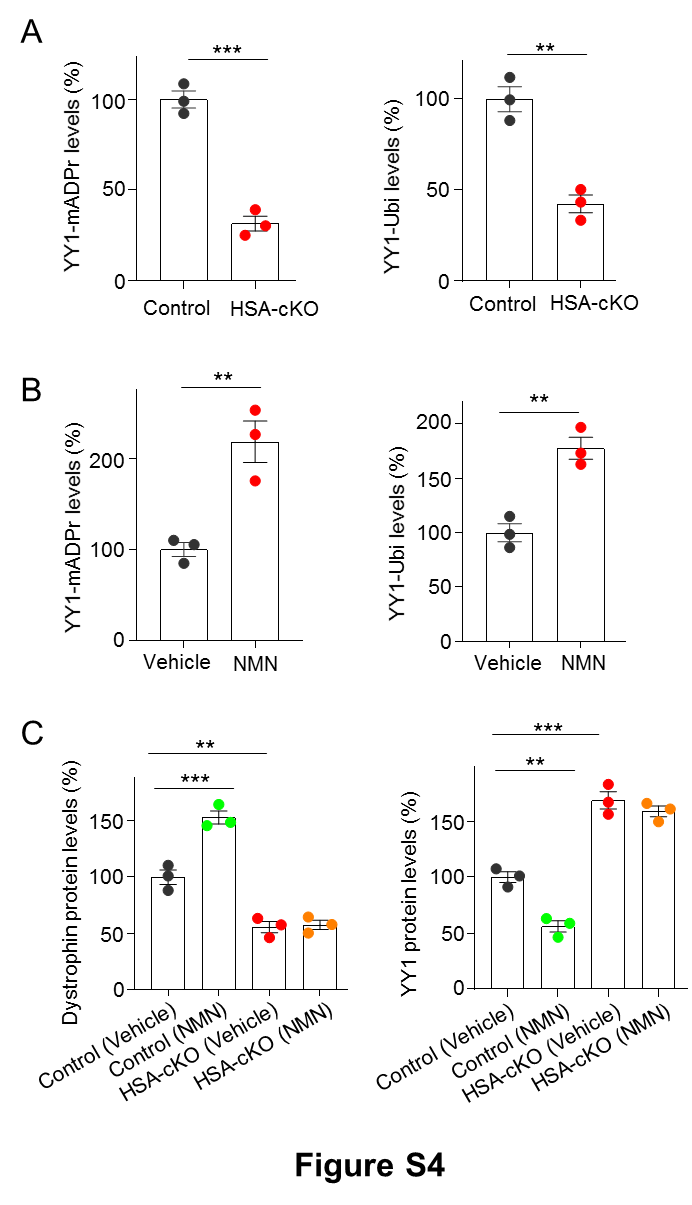
**

**Figure S4. Sirt6 mono-ADP-ribosylates YY1 to promote Dystrophin expression (relates to Figures 5 and 6).**

(A) Statistical results of Figure 5G.

(B) Statistical results of Figure 6C.

(C) Statistical results of Figure 6D. One-way ANOVA with Tukey's multiple comparisons test. Dystrophin: F (3, 8) = 71.28, YY1: F (3, 8) = 85.12.

Unless otherwise specified, at least three independent experiments were performed. Mean ± SEM; **p < 0.01; ***p < 0.001; t-test in (A and B), one-way ANOVA in (C).

**Table S1 Primer sequences used in this study were as follows:**

| **Primer** | **Sequence (5' -> 3')** |
| --- | --- |
| Sirt6-F (for GT) | AGTGAGGGGCTAATGGGAAC |
| Sirt6-R (for GT) | AACCCACCTCTCTCCCCTAA |
| HSA-cre-F(for GT) | GCCTGCATTACCGGTCGATGCAACGA |
| HSA-cre-R(for GT) | GTGGCAGATGGCGCGGCAACACCATT |
| Sirt6-F | ATGTCGGTGAATTATGCAGCA |
| Sirt6-R | GCTGGAGGACTGCCACATTA |
| MyoD-F | ATGATGACCCGTGTTTCGACT |
| MyoD-R | CACCGCAGTAGGGAAGTGT |
| MyoG -F | GAGACATCCCCCTATTTCTACCA |
| MyoG -R | GCTCAGTCCGCTCATAGCC |
| Dystrophin-F | CGAGACCCAAACCACTTGTTG |
| Dystrophin-R | GGTCAGCTAAAGACTGGTAGAGC |
| Utrophin-F | ATTTTGCCAAACATCCTCGGC |
| Utrophin-R | GACTGGGAGGGGTCATAGTG |
| α-dystrobrevin1-F | TCCCGGAAACCTGGGTACATT |
| α-dystrobrevin1-R | CATCCTCGGGTTGCGTGTAA |
| α-dystrobrevin2-F | TTGGAACGTCATTGAAGCATTGC |
| α-dystrobrevin2-R | GCATCCTCTTGTTGAGCTGGT |
| α-syntrophin-F | GGGGCCGGGAAAACAAGAT |
| α-syntrophin-R | ATCCCCAACAAAAAGGGCCTC |
| β1-syntrophin-F | AACAGGCAGCTAGAAATTCACTC |
| β1-syntrophin-R | AAGTCACCAGCGTTGGAATGA |
| β2-syntrophin-F | CCCTCTCAAAATGTGCTTTGC |
| β2-syntrophin-R | GCAGCGTAAGATCAAGGTGTT |
| γ1-syntrophin-F | TCCCTCCAAGACCGTGTGTAT |
| γ1-syntrophin-R | CCTTGAGAACCTTGCACATGAT |
| γ2-syntrophin-F | GGGCGTCCTAGTCACCTACT |
| γ2-syntrophin-R | GCACCTCTTTTGTCAGCTTCA |
| Dystroglycans-F | CTTGAGGCGTCCATGCACT |
| Dystroglycans-R | GGCAATTAAATCCGTTGGAATGC |
| SGCA-F | ACTTCCGCGTTGACTGGTGCA |
| SGCA-R | CACCAAGGCATCTGTCAGGAAG |
| SGCB-F | GGCAACTTAGCCATCTGCGTGA |
| SGCB-R | GTGGAACTCCATGCTATCACACC |
| SGCD-F | TGAGACTGGAGTCCAAGGATGG |
| SGCD-R | CTCGAAGACCTTCTGCCTCGTT |
| SGCG-F | GTGACAGTCAGTGCTCGCAACT |
| SGCG-R | GCAGAGAACAGTGGCTTGCCAT |
| eNOS-F | GTTTGTCTGCGGCGATGT |
| eNOS-R | GTGCGTATGCGGCTTGTC |
| YY1-F | CAGTGGTTGAAGAGCAGATCAT |
| YY1-R | AGGGAGTTTCTTGCCTGTCAT |
| Chrna1-F | CTCTCGACTGTTCTCCTGCTG |
| Chrna1-R | GTAGACCCACGGTGACTTGTA |
| Chrnb1-F | CTCCAACTATGATAGCTCGGTGA |
| Chrnb1-R | CAGGTCTAAGTACACCTTTGTGC |
| Chrng-F | CATCTCCTCAGTCGCCATCC |
| Chrng-R | CACGACCACAGAGTTCACGA |
| Chrnd-F | GAATGAGGAACAAAGGCTGATCC |
| Chrnd-R | GGTGAGACTTAGGGCGACAT |
| Chrne-F | CTATTTCCCCTTTGACTGGC |
| Chrne-R | CCTCCCTCATAGCGGCGAAT |
| Gapdh-F | ATGGTGAAGGTCGGTGTGAAC |
| Gapdh-R | AGTGGAGTCATACTGGAACATG |
| Dystrophin-F (ChIP) | AGTTGAACATTATTTGAACT |
| Dystrophin-R (ChIP) | ACTGAGTGAGTCAACATAGT |
| Sirt6-sgRNA | AATGTGGCAGTCCTCCAGCG |
| YY1-sgRNA | CGACCCGGGGAATAAGAAGT |
